# Development of Sulfadiazine-Decorated PLGA Nanoparticles Loaded with 5-Fluorouracil and Cell Viability

**DOI:** 10.3390/molecules20010879

**Published:** 2015-01-08

**Authors:** Pedro Pires Goulart Guimarães, Sheila Rodrigues Oliveira, Gabrielle de Castro Rodrigues, Savio Morato Lacerda Gontijo, Ivana Silva Lula, Maria Esperanza Cortés, Ângelo Márcio Leite Denadai, Rubén Dario Sinisterra

**Affiliations:** 1 Chemistry Department, Institute of Exact Sciences, Universidade Federal de Minas Gerais, Av. Antonio Carlos, 6627, Pampulha, CEP 31270-901 Belo Horizonte-MG, Brazil; E-Mails: pedropiresg@ufmg.br (P.P.G.G.); shetq@yahoo.com.br (S.R.O.); gabrielledecastro@hotmail.com (G.C.R.); ivanalula@ufmg.br (I.S.L.); 2 Department of Restorative Dentistry, Faculty of Dentistry, Universidade Federal de Minas Gerais, Av. Antonio Carlos, 6627, Pampulha, CEP 31270-901 Belo Horizonte-MG, Brazil; E-Mails: savio.morato@yahoo.com.br (S.M.L.G.); mecortes@yahoo.com (M.E.C.); 3 Pharmaceutical Department, Universidade Federal de Juiz de Fora, Campus Governador Valadares-MG, Av. Dr. Raimundo Monteiro de Rezende, 330, Centro, CEP 35010-177 Governador Valadares-MG, Brazil; E-Mail: angelo.denadai@ufjf.edu.br

**Keywords:** 5-FU, PLGA, antitumor nanoparticles, sulfadiazine, drug delivery

## Abstract

The aim of this work was to synthesize sulfadiazine-poly(lactide-co-glycolide) (SUL-PLGA) nanoparticles (NPs) for the efficient delivery of 5-fluorouracil to cancer cells. The SUL-PLGA conjugation was assessed using FTIR, ^1^H-NMR, ^13^C-NMR, elemental analysis and TG and DTA analysis. The SUL-PLGA NPs were characterized using transmission and scanning electron microscopy and dynamic light scattering. Additionally, the zeta potential, drug content, and *in vitro* 5-FU release were evaluated. We found that for the SUL-PLGA NPs, D_h_ = 114.0 nm, ZP = −32.1 mV and the encapsulation efficiency was 49%. The 5-FU was released for up to 7 days from the NPs. Cytotoxicity evaluations of 5-FU-loaded NPs (5-FU-SUL-PLGA and 5-FU-PLGA) on two cancer cell lines (Caco-2, A431) and two normal cell lines (fibroblast, osteoblast) were compared. Higher cytotoxicity of 5-FU-SUL-PLGA NPs were found to both cancer cell lines when compared to normal cell lines, demonstrating that the presence of SUL could significantly enhance the cytotoxicity of the 5-FU-SUL-PLGA NPs when compared with 5-FU-PLGA NPs. Thus, the development of 5-FU-SUL-PLGA NPs to cancer cells is a promising strategy for the 5-FU antitumor formulation in the future.

## 1. Introduction

Colorectal cancer is one of the main types of diagnosed cancer and more than half a million patients may die from the disease each year [1]. Over the last decade, significant progress in chemotherapy for colorectal cancer has been made [2]. Among the drugs that have been used in colorectal cancer, 5-fluorouracil (5-FU) is still one of the most active anti-colorectal cancer drugs [2,3,4]. 5-FU can exert cytotoxic effects through the inhibition of thymidylate synthetase or through its incorporation into RNA and DNA via its intracellular metabolites, both of which ultimately activate apoptosis in targeted cancer cells [5]. However, anticancer agents, such as 5-FU, are frequently associated with systemic toxicity because they are not only potent killers of cancer cells but also of normal cells [6,7]. Furthermore, their short *in vivo* half-life means that a sustained therapeutic effect cannot be obtained [8].

Functionalized polymeric systems, such as nanoparticles (NPs), are promising carriers for targeted anticancer drug delivery because these systems are preferentially internalized by diseased cells by the phenomenon of enhanced permeability and retention (EPR), therefore leading to better overall efficiency of the therapeutic agent [9,10,11,12,13,14,15,16].

Poly(lactide-co-glycolide) (PLGA) has been extensively used as a polymeric system, due to its biocompatibility, biodegradability and its approval by the U.S. Food and Drug Administration (FDA) for human intravenous, oral, and dermal applications [17,18,19,20]. Many studies have been conducted to improve the drug circulation time and tumor targeting of PLGA NPs [7,9,10,14,18,20]. For example, for nanosystems to penetrate the tumor and be effective in cancer treatment, the ideal dimensions would be in the range of 10–100 nm because anything smaller might be filtered out and then removed by the kidneys and larger particles could be taken up by macrophages [21].

The success of nanosystems is related to the careful selection of targeting moieties, small size and surface properties, which would ideally provide a high selectivity of the NPs [22,23,24,25,26,27,28,29,30]. The most commonly used types of ligands include monoclonal antibodies, aptamers, cell specific peptides, carbohydrates and small molecules [31,32].

In this study, sulfadiazine (SUL) was chosen as the ligand for the PLGA polymer because studies have reported that SUL and its derivatives have the capacity to concentrate in tumor cells, such as Walker carcinoma and Yoshida sarcoma, where the SUL concentration was found to be two to three times higher than that in the liver [33,34,35]. Moreover, other researchers have shown that SUL enhances the activity of anticancer drugs against tumor cells [34,36,37,38,39] and sulfonamide derivative ligands have shown antitumor activity [34,36,37,38,40,41] by several mechanisms such as angiogenesis matrix metalloproteinase inhibition, cell cycle perturbation in the G1 phase and disruption of microtubule assembly [42,43,44].

Thus, we developed a novel SUL-PLGA decorated NPs based on the SUL ligand and the acid terminated PLGA in order to obtain an efficacious deliver of the antitumor drug 5-FU. It was hypothesized in this work that the SUL presence could enhance the cytotoxicity of the NPs loaded with 5-FU against cancer cells and decrease the cytotoxicity against normal cells.

First of all, the SUL-PLGA conjugation was assessed using FTIR, ^1^H-NMR, ^13^C-NMR, elemental analysis and TG and DTA analysis. Then, the SUL-PLGA NPs were produced and characterized using transmission and scanning electron microscopy and dynamic light scattering. Additionally, the zeta potential, drug content, *in vitro* 5-FU release and cytotoxicity on cancer cell lines and normal cell lines were evaluated.

## 2. Results and Discussion

### 2.1. PLGA Polymer Chemical Modification with SUL

**Scheme 1:**
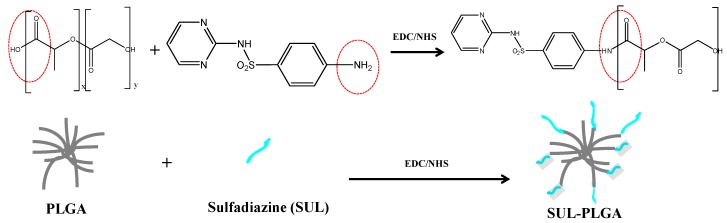
Conjugation reaction of PLGA–COOH with sulfadiazine carbodiimide and N-hydroxysuccinimide.

SUL-PLGA was synthesized by the direct conjugation of the PLGA–COOH carboxyl groups with the amine group of SUL according to the coupling chemistry described in Scheme 1. Briefly, the PLGA–COOH carboxyl groups reacts with EDC and NHS forming a succinimide ester, which undergoes nucleophilic attack of the nonbonding electron pair of the nitrogen atoms of the amino group of sulfadiazine to form the amide bond [45,46]. The synthesis of the SUL-PLGA conjugates was confirmed using FTIR, ^1^H-NMR, ^13^C-NMR and thermal analysis as discussed below.

### 2.2. Characterization of the SUL-PLGA Conjugates Using Thermal Analysis

TGA and DTA analyses of PLGA, SUL, SUL/PLGA physical mixture and SUL-PLGA were performed and compared to investigate the thermal behavior of the compounds in the solid state (Supplementary Material). The TGA curves of all compounds tested showed different decomposition profiles. First, PLGA exhibited thermal stability up to 67 °C and no residue formation during the heating of this compound (TGA curve). In the DTA curve, there was an endothermic peak at 45.3 °C related to the glass transition temperature of PLGA, and this transition was consistent with those previously described in the literature [47]. The SUL TGA profile curve showed thermal stability up to approximately 260 °C, and this result may be due to the inter hydrogen bonding interaction between the -NH moieties of the SUL molecules, followed by molecule thermodecomposition forming a final residue of 33%. This observation corresponds to an endothermic fine peak at approximately 260 °C observed in the DTA curve, which was attributed to the simultaneous melting and thermodecomposition of SUL.

A lower thermal stability of the SUL-PLGA modified polymer was observed in the TGA curve in comparison with the SUL/PLGA physical mixture. Initially, a weight loss of approximately 20% was observed from 60.8 °C up to 272.9 °C and attributed to the dehydration and partial decomposition of the compound. A similar event was not observed in the TGA of the SUL/PLGA physical mixture. A second thermo decomposition process of SUL-PLGA of approximately 79% was observed in the temperature range of 272.9 to 394.7 °C and attributed to the thermal decomposition of PLGA and SUL. Finally, a lower residue of SUL-PLGA (1%) was verified when compared with SUL/PLGA physical mixture (14%) and SUL (33%).

In the DTA curves of the SUL/PLGA physical mixture and SUL-PLGA, no endothermic events associated with the melting of SUL or the glass transition temperature of PLGA were observed. Thus, the modification of PLGA with SUL was able to change the thermal properties of the compound.

### 2.3. Amide Bond Detection Using FTIR

The covalent conjugation of SUL to PLGA via the formation of an amide bond was confirmed by observing a strong band at 1714 cm^−1^ and a weak band at 1624 cm^−1^ corresponding to the amide I and amide II bands, respectively (Supplementary Material). These amide I and II bands were not observed in either the PLGA or SUL spectra. The band at 3500–3350 cm^−1^ is related to ν_OH_ stretching, which confirms the structure of PLGA, and the medium intensity band at 3350 cm^−1^ is related to ν_N-H_ stretching from the conjugated SUL [38]. Additionally, we observed intensified bands at 2934 and 2854 cm^−1^ related to the symmetric and asymmetric stretches of ν_s_, ν_assCH2_ and ν_s_, ν_assCH3_ when compared to the PLGA spectrum due to the overlapping of these vibrational modes of PLGA and SUL. A characteristic stretching band of ν_C=C_ from the SUL pyrimidine ring at 1582 cm^−1^ and in the region between 1214 and 1092 cm^−1^ verified an overlap of the absorption bands of symmetrical and asymmetrical stretching (ν_s_, ν_assSO2_) from SUL characteristic bands from PLGA and the reduction in the intensity of the bands may be related to the decrease of free carboxyl groups after the reaction (Supplementary Material).

### 2.4. ^1^H-NMR and ^13^C-NMR

The syntheses of the SUL-PLGA conjugates were confirmed using ^13^C-NMR and ^1^H-NMR (Supplementary Material). The hydrogen signals of the SUL aromatic region (δ 7.0–8.0 ppm), the hydrogen signals at δ 5.2 and δ 1.6 ppm, which originate from the -CH- protons and -CH_3_ protons of the PLA block, and the hydrogen signal at δ 4.8 ppm, which belongs to the -CH_2_- protons of the PGA block, were observed in the SUL-PLGA ^1^H-NMR spectra. In addition, in the ^13^C-NMR spectra, the PLGA peaks at δ 16.7 ppm (C, CH_3_), δ 60.7 (C, CH_2_), and δ 69.04 ppm (C, CH) were as described in the literature [48]. Furthermore, SUL and PLGA signals were observed in the region after δ 155 ppm, namely, at δ 159.4 ppm (C, aromatic ring of SUL), δ 166.4 ppm (C, pyrimidine ring of SUL) [49], δ 169.5 ppm (C, C=O of PLGA) and δ 173.1 ppm (C, from amide bond) confirming the SUL-PLGA modified polymer.

### 2.5. SUL Functionalization Degree (FD) on the Carboxylic Groups of PLGA

The FD of the PLGA carboxylic group to amide groups, assessed by elemental analysis and ^13^C-NMR were 29% and 21%, respectively. Two peaks were selected in the ^13^C-NMR spectra, at δ 16.7 ppm (C, CH_3_ PLGA) and δ 166.4 ppm (C, pyrimidine ring of SUL), and both were integrated. The peak assigned to sulfadiazine had a value of δ 0.42 (0.21 to each identical carbon), and PLGA showed a value of δ 1.00 (Supplementary Material).

### 2.6. Size, Morphology and Surface Charge Measurements of NPs

**Figure 1:**
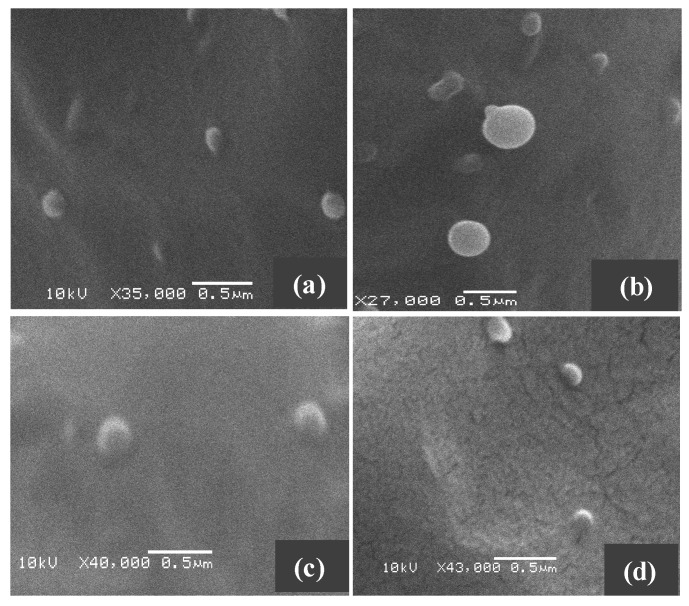
Representative scanning electron microscopy image of (**a**) 5-FU-SUL-PLGA; (**b**) SUL-PLGA; (**c**) 5-FU-PLGA; (**d**) PLGA.

The surface morphology and size of PLGA, SUL-PLGA, 5-FU-PLGA and 5-FU-SUL-PLGA NPs before and after the drug encapsulation process were evaluated using scanning electron microscopy (SEM). The image shown in Figure 1 demonstrates that the NPs were dispersed as individual particles with a well-defined spherical surface and relatively homogeneously distributed around a size of 100–200 nm range, suggesting that neither FU encapsulation nor the SUL substitution process are able to cause significant morphological differences among the four NPs.

Using DLS, a similar average hydrodynamic diameter (D_h_) of the drug-loaded NPs and a low polydispersity were observed (Table 1). Also, the changes on D_h_ between four groups of NPs were not significant when considered the statistical analysis carried out using ANOVA.

For the PLGA and SUL-PLGA NPs (the control systems), the sizes were 137.1 ± 8.6 nm and 105.6 ± 13.9 nm, respectively, and the zeta potentials were −39.1 ± 5.5 mV and −37.3 ± 1.3 in deionized water, respectively. For 5-FU-PLGA and 5-FU-SUL-PLGA, the sizes were 133.1 ± 16.9 and 114.6 ± 14.6, respectively, and the zeta potentials were −39.3 ± 0.7 mV and −32.1 ± 2.14 mV in deionized water, respectively. The zeta potentials values that were verified here help to avoid nanoparticle aggregation. Also, it is disclosed in the literature that the negative surface charge plays an important role in the biocompatibility and cellular uptake of the NPs [50,51,52,53,54,55]. For example, Patil *et al.* found a higher cellular uptake and lower protein adsorption of cerium oxide NPs with a negative charge compared with positively charged NPs [53]. On the other hand, others studies also reported that positively charged NPs are taken-up by cells more efficiently [56]. In addition, negatively charged NPs also have additional advantages compared to positively charged particle, such as, lower induction of inflammation than positively charged particle [50,57], lower induction of the T-cell proliferation and cytokine production and secretion than a cationic surface charge [50], and less damage to the erythrocyte membranes than positively charged particle [58].

**Table 1:** Characterization of PLGA and SUL-PLGA drug-loaded NPs.

Groups	Particle Size (nm)	Polydispersity	Zeta Potential (mV)
PLGA	137.1 ± 8.6	0.091 ± 0.003	−39.1 ± 5.5
5-FU-PLGA	133.1 ± 16.9	0.099 ± 0.025	−39.3 ± 0.7
SUL-PLGA	105.6 ± 13.9	0.133 ± 0.023	−37.3 ± 1.3
5-FU-SUL-PLGA	114.6 ± 14.6	0.112 ± 0.018	−32.1 ± 2.14

### 2.7. Drug Loading

The amount of free 5-FU in the supernatant was determined using a UV-vis analysis. The encapsulation efficiency (EE) and drug loaded content (LC) values were calculated according to equations described in the Supplementary Material.

The encapsulation efficiencies (EE) and drug loaded content of 5-FU-PLGA and 5-FU-SUL-PLGA were all similar, as reported in Table 2. These data suggest that the 5-FU shows the same affinity for the PLGA and PLGA-SUL matrices. Ocal *et al.* achieved higher encapsulation efficiencies of 5-FU-loaded NPs, around 83.6%–93.9%, than showed in this work [59]. However, in that system, the nanoparticles were formed with hydrophobic core polymer and triblock copolymers; Poly(D, L-lactic acid), Poly(ethylene glycol)-block-poly(propylene glycol)-block-poly(ethylene glycol) copolymer (PLA/PEG-PPG-PEG) and Poly(D, L-lactide-co-glycolide)/Poly(ethylene glycol)-block-poly(propylene glycol)-block-poly(ethylene glycol) copolymer (PLGA/PEG-PPG-PEG).

**Table 2:** Effects of formulation parameters on loading content of drugs and encapsulation efficiency of NPs.

Groups	Encapsulation Efficiency (EE) (%)	Loaded Content (LC) (%)
5-FU-PLGA	48.7 ± 5.7	12.7 ± 1.3
5-FU-SUL-PLGA	48.9 ± 4.4	12.8 ± 1.0

### 2.8. Differential Scanning Calorimetry (DSC) Measurements

**Figure 2:**
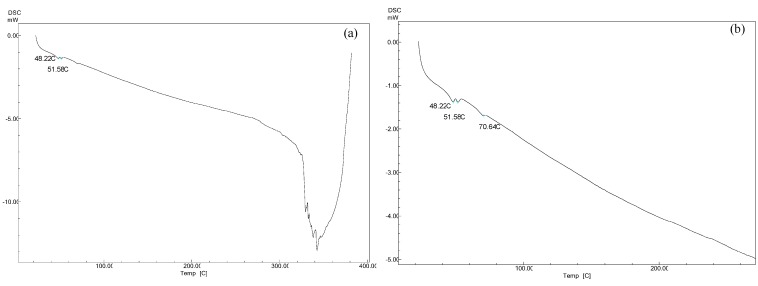
DSC curves 5-FU-loaded SUL-PLGA NPs: (**a**) DSC curves heated from 25 °C to 400 °C; (**b**) DSC curves with zoom up to 300 °C.

The DSC curves for PLGA and 5-FU-loaded SUL-PLGA NPs are shown in Figure 2. The 5-FU endothermal peak at 282–286 °C associated with its melting point was not observed in 5-FU-loaded NPs, which indicates that no crystalline 5-FU was found outside of the NPs [60]. The similar melting transition properties of the loaded and unloaded NPs show that the SUL-PLGA polymer remained unaffected during encapsulation.

### 2.9. 5-FU Release Kinetics

The study of the *in vitro* release kinetics of a drug encapsulated into NP carriers is important to check the stability and efficiency of 5-FU controlled release.

**Figure 3:**
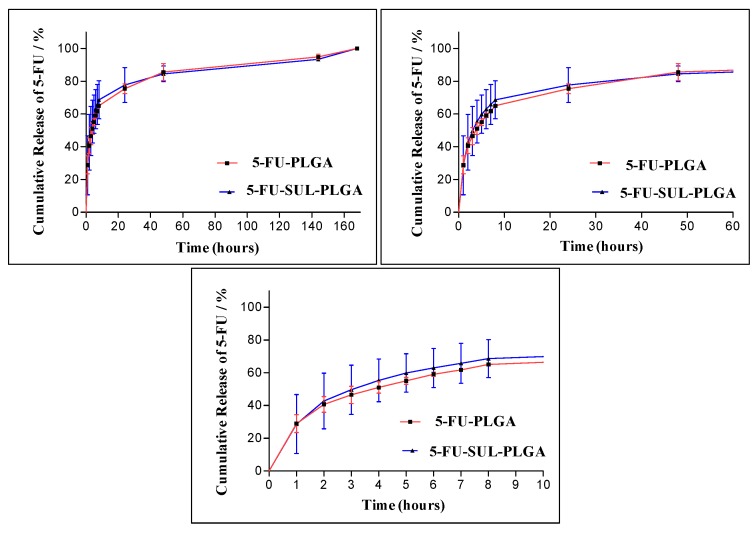
Release profiles of 5-FU from 5-FU-PLGA and 5-FU-SUL-PLGA in different times (vertical bar means average ± standard deviation, *n* = 3).

In this study, a significant burst effect from the 5-FU-PLGA and 5-FU-SUL-PLGA NPs was observed with approximately 60% and 63%, respectively, released up to 8 h (Figure 3). During a seven-day incubation period, the complete release of 5-FU was verified from both 5-FU-PLGA and 5-FU-SUL-PLGA NPs. Thus, the surface modification of the PLGA NPs with SUL did not reduce the burst release of 5-FU relative to undecorated SUL NPs (Figure 3).

Using the 5-FU drug release data from the 5-FU-PLGA and 5-FU-SUL-PLGA NPs, we applied four release models to obtain more insight into the 5-FU release kinetics. The models used were the Higuchi model [61,62,63,64], the zero order model, the first order model and the Hixson-Crowell model [62,65]. The respective values for the correlation coefficients (R_c_) calculated for each model are shown in Table 3. The figures showing the linearization of the release curves obtained for the NPs after the burst effect (first 8 h) up to 168 h according to the models are available in the Supplementary Material.

**Table 3:** Correlation coefficients (Rc) and *p* calculated for different models of drug release kinetics.

Model	Higuchi		Hixson-Crowell		Zero Order		First Order	
NPs	R_c_	*p*	R_c_	*p*	R_c_	*p*	R_c_	*p*
5-FU-PLGA	0.8434	<0.0001	0.5258	0.0076	0.7020	<0.001	0.5407	0.0064
5-FU-SUL-PLGA	0.7890	0.0001	0.4891	0.0114	0.6475	0.0016	0.4486	0.0172

A comparison of the R_c_ values shows that the NPs exhibit controlled release by diffusion through pores, which are formed during the degradation of the NPs, and are better modeled by the Higuchi model, which maintains a nearly constant release with the fraction of drug released varying linearly with the square root of time and it is in accordance with literature [59]. According to [62], the curves modeled by Higuchi have a low release rate because this model uses Fick’s first law, in which the flow of drug from a matrix is primarily directed by diffusion, with a slow drug release to the external environment.

**Figure 4:**
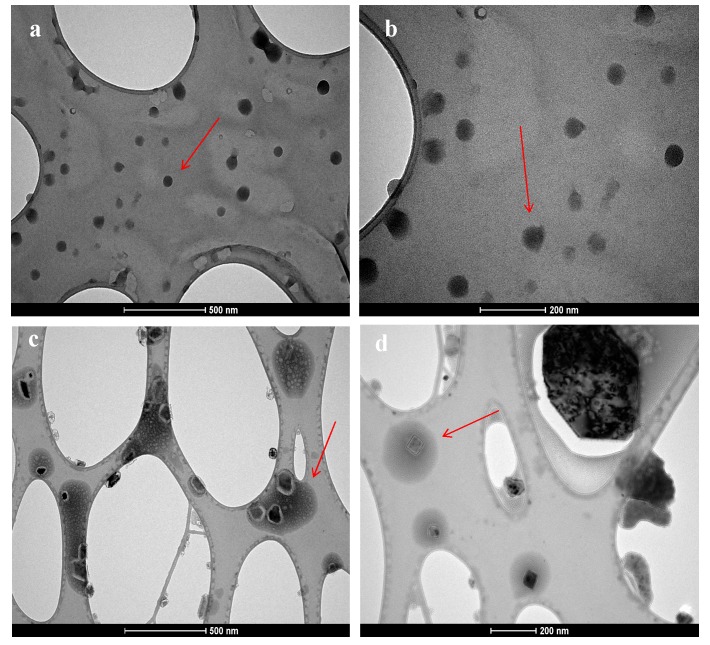
TEM of 5-FU-SUL-PLGA NPs at different degradation states. Immediately after preparation (**a**,**b**); after 7 days (**c**,**d**), in releasing medium at 37 °C.

Furthermore, the degradation of the PLGA and SUL-PLGA NPs might be a critical factor in determining the release of 5-FU from these matrices; thus, the degradation of the NPs under the conditions used occurs by the simple hydrolytic cleavage of ester groups. The morphology and size studies using transmission electron microscopy (TEM) supported a mechanism of homogenous degradation of the NPs, showing the progressive formation of pores and irregular surface areas all over the NPs matrix and an increase in particle size, as shown in Figure 4c,d.

### 2.10. Acid-Base Titration and Zeta Potential (ZP) Measurements

**Figure 5:**
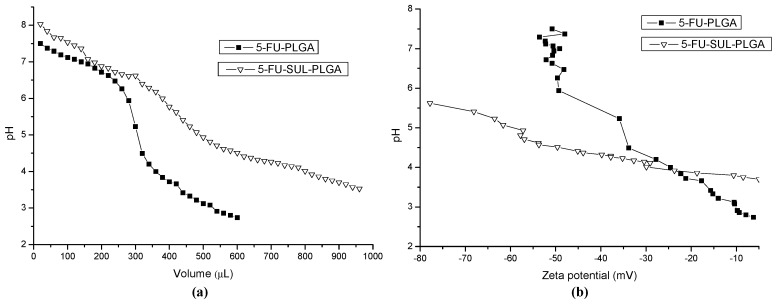
(**a**) Acid–base titration curves of 5-FU-PLGA and 5-FU-SUL-PLGA suspensions; (**b**) Zeta potential measures by Acid–base titration curves of 5-FU-PLGA and 5-FU-SUL-PLGA suspensions.

The pH titration and ZP measurements shown on Figure 5a,b were performed in order to investigate the influence of pH dependence on 5-FU-PLGA and 5-FU-SUL-PLGA NPs and the buffering property of the NPs. Initially, the 5-FU-SUL-PLGA NPs showed higher negative zeta potential (−80 mV) compared with the 5-FU-PLGA NPs (−50 mV). Follow, it was observed a higher decreasing of the ZP values and pH from 8 to 5 of 5-FU-PLGA NPs, most likely caused by the protonation of PLGA acid group, suggesting a higher aggregation process, and higher hydrophobicity. In contrast, the 5-FU-SUL-PLGA suffered slightly decreasing ZP values and pH from 6 to 5 not only by the protonation of PLGA acid group, but also by the protonation of amine groups from sulfadiazine linked at the PLGA surface. These results suggest higher colloidal stability of 5-FU-SUL-PLGA which could provide additional protection to NPs when circulate in blood upon administration.

### 2.11. Cytotoxicity Test

**Figure 6:**
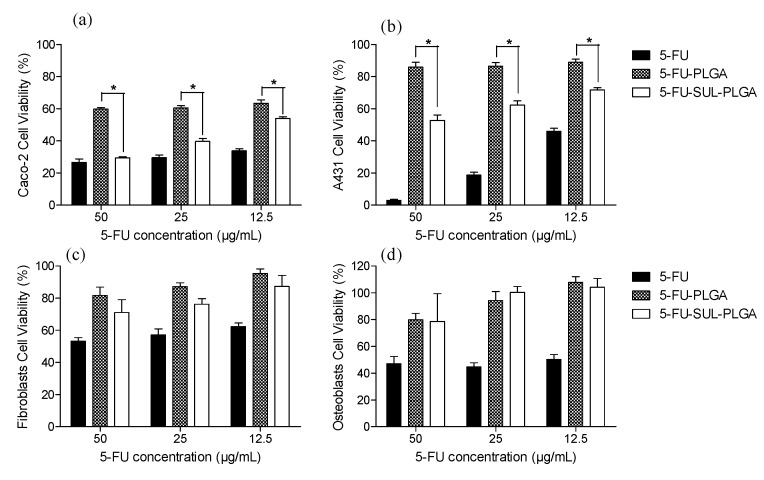
Cytotoxicity of free 5-FU, 5-FU-PLGA and 5-FU-SUL-PLGA on: (**a**) Caco-2 cells; (**b**) A431 cells; (**c**) fibroblast cells and (**d**) osteoblast cells at different 5-FU concentrations (50, 25 and 12.5 µg/mL) (*p* < 0.05). * The difference is statistically significant.

The cytotoxicity of the various 5-FU formulations (free 5-FU, 5-FU-PLGA and 5-FU-SUL-PLGA) against Caco-2 cells at distinct drug concentrations (50, 25 and 12.5 µg/mL of 5-FU) was compared (Figure 6a). A cytotoxicity approximately 1.6-fold higher was observed with the 5-FU-SUL-PLGA NPs compared to the 5-FU-PLGA NPs at all concentrations. These results suggest that the presence of SUL could significantly enhance the cytotoxicity of 5-FU-SUL-PLGA NPs compared to the 5-FU-PLGA NPs.

A lower cytotoxicity for the 5-FU-SUL-PLGA NPs against A431 cells was observed compared with that observed against the Caco-2 cells (Figure 6b). In contrast, a higher cytotoxicity was verified for free 5-FU against the A431 cells compared with the Caco-2 cells (Figure 6a,b). Furthermore, a 1.5-fold increased cytotoxicity against A431 cells was observed for the 5-FU-SUL-PLGA NPs compared with the 5-FU-PLGA NPs.

Interestingly, a similar effect was observed for 50 µg/mL free 5-FU and the 5-FU-SUL-PLGA NPs against Caco-2 cells. In contrast, free-FU had a higher cytotoxicity against A431 cells compared to 5-FU-SUL-PLGA NPs at all concentrations (Figure 6b).

Analyzing the cytotoxicity of 5-FU-SUL-PLGA and the 5-FU-PLGA NPs against fibroblast and osteoblast cells it can be observed significant differences when compared to cancer cells (Figure 6c,d, respectively).

Surprisingly, the 5-FU-SUL-PLGA NPs had lower cytotoxicity against fibroblast and osteoblast cells (normal cells) than against cancer cells (Caco-2 and A431) and when compared to free 5-FU drug (Figure 6). The low or negligible cytotoxic effects on the normal cells are highly desirable to avoid possible mutagenic effects in these cells when exposed to an anticancer drugs for a long time [66]. These results can be explained as a consequence of two synergic effects related in the literature for the SUL namely the cancer cell concentration and its pH dependence which increase the SUL hydrophobicity and thus the cytotoxicity [33,34,35,67]. These results suggest that the SUL conjugated NPs enhance the cytotoxicity against cancer cells by the SUL preferential concentration relative to fibloblast and osteoblast cells. In addition, it is possible that there is a higher cancer cell-NP interaction through non-specific hydrogen bonding between SUL and the cellular membrane, which is composed of glycosaminoglycans and proteoglycans that help NPs adhere to the cell membranes.

These results could be very important as fibroblasts are involved in the extracellular matrix (ECM) formation and collagen and elastin production, and the preservation of these cells against drugs is of great importance [66]. Furthermore, fibroblasts are associated with the dynamic remodeling of the ECM that is essential for development, wound healing and normal organ homeostasis. In addition, the ECM could contribute to the remodeling of fibrotic diseases and cancer when stimulated by external agents. Fibroblasts can also differentiate into other types of pathologic cells in response to pathogenic stimulus [68].

We report in this study that osteoblasts were not affected by various concentrations of 5-FU-SUL-PLGA NPs, thus leaving such cells free from the effect of chemotherapeutic drugs and maintaining the potential for regeneration and continual remodeling throughout life. From this perspective, the biggest challenge with respect to clinical treatment is to treat only the cancer cells and we focused on the higher compatibility of 5-FU-SUL-PLGA NPs with fibroblasts and osteoblasts and its higher cytotoxicity on cancer cells. Both types of normal cells can be present in the tissues surrounding the tumor or even associated with the differentiation of other types of cells. Thus, if a drug has an effect on normal cells, it could further damage the pathological condition.

## 3. Experimental Section

### 3.1. Reagents and Materials

All reagents were purchased and used as received without further purification. The 1-ethyl-3-(3-dimethylaminopropyl) carbodiimide (EDC) and *N*-hydroxysuccinimide (NHS) were purchased from Sigma Chemical Company (St. Louis, MO, USA), 50/50 Poly(lactide-co-glycolide) acid terminated (PLGA), molecular weight 43.9 kDa, from Lactel Absorbable Polymers (Denver, CO, USA), Sulfadiazine (SUL) from Gênix Indústria Farmacêutica Ltda. (Anápolis, Brazil) and 5-fluorouracil (5-FU) from Araújo Supplies (Belo Horizonte, Brazil). For the cell culture experiments, the human epithelial colorectal adenocarcinoma cell line Caco-2 (ATCC HTB-37) was generously donated by Pontificia Universidad Javeriana (Bogotá, Colombia), the human epidermoid carcinoma cell line A431 (ATCC CRL-1555) was generously donated by Universidade Federal de Minas Gerais (Belo Horizonte, MG, Brazil), and the human fibroblasts (ATCC CL-173) were purchased from Associação Técnico Científica Paul Ehrlich (Rio de Janeiro, RJ, Brazil). The Dulbecco’s Modified Eagle’s Medium (DMEM), high and low glucose, fetal bovine serum (FBS), penicillin–streptomycin (pen–strep), and trypsin/ethylenediamine tetraacetic acid (trypsin-EDTA) were obtained from Invitrogen (New York, NY, USA). The 3-(4,5-Dimethylthiaol-2-yl)-2,5-diphenyltetrazolium bromide (MTT) was obtained from Sigma-Aldrich (Saint Louis, MO, USA). The collagenase type II and 4-(2-hydroxyethyl)-1-piperazineethanesulfonic acid (HEPES) were obtained from Gibco (Paisley, Scotland, UK). The sodium dodecyl sulfate (SDS) was obtained from LGC Biotecnologia (São Paulo, SP, Brazil), and the L-glutamine was obtained from Vetec (Rio de Janeiro, RJ, Brazil).

### 3.2. PLGA Polymer Chemical Modification with SUL

SUL-PLGA was synthesized through a chemical modification method previously reported in the literature [45,46]. Briefly, PLGA (1 g) dissolved in acetone (15 mL) was activated by EDC (159.62 mg) and NHS (88.5 mg) at room temperature (25 ± 2 °C) under a nitrogen atmosphere for 24 h. The activated PLGA was added slowly to SUL (110 mg) dissolved in acetone (15 mL) in a dropwise manner with gentle stirring. The reaction was carried out for 6 h at room temperature, and the resultant solution was completely dried under a vacuum. Thereafter, the product SUL-PLGA was washed three times with 0.1 mol·L^−1^ aqueous hydrochloric acid (HCl) solution to remove excess reagent, such as EDC, NHS and sulfadiazine. Then, SUL-PLGA was purified by dialysis method using a semi-permeable membrane 3500 Da MWCO for four days.

### 3.3. Solid State Analysis

The solid-state characterization of PLGA, SUL-PLGA and SUL/PLGA physical mixture was performed using TGA, DTA, differential scanning calorimetry (DSC) and Fourier-transform infrared spectroscopy (FTIR). The TGA and DTA analyses were performed in duplicate using a TGA/DTA modulus (SDT Q600, TA Instruments, Lindon, UT, USA). Briefly, the samples (~5 mg) were placed on open pans of aluminium oxide (Al_2_O_3_) and heated from ambient temperature (~25 °C) to 700 °C at a rate of 10 °C min^−1^. The sensitivity was 1.0 °C, and the nitrogen gas flow rate was 50 mL·min^−1^. For FTIR, the samples were prepared as potassium bromide (KBr) pellets, and scans were performed from 4000 to 400 cm^−1^ at a resolution of 4 cm^−1^ using 32 scans per sample. The FTIR spectra were recorded using a Perkin Elmer spectrometer (Spectrum GX; Perkin Elmer, Boston, MA, USA) to confirm the chemical conjugation of SUL to PLGA. Differential Scanning Calorimetry (DSC) was carried out using a DSC60 instrument (Shimadzu, Kyoto, Japan) to investigate the thermal property of 5-FU inside the NPs. Finally, 5 mg 5-FU, PLGA and 5-FU-loaded SUL-PLGA NPs were weighted and placed onto standard aluminum pans (2 mm high & 4 mm diameter). The samples were cooled to −80 °C and heated from 25 °C to 400 °C with a heat flow rate of 10 °C·min^−1^. The calibration was carried out using an aluminum pan as a reference.

### 3.4. Nuclear Magnetic Resonance (NMR) Spectroscopy

The SUL-PLGA in solution was characterized using NMR spectroscopy. ^1^H-NMR and ^13^C-NMR chemical shift (δ) experiments were performed using a Bruker DPX-400 Avance (400 MHz) spectrometer (Bruker, Rheinstetten, Germany) at 300 K. The solutions analyzed included 2.0 mM PLGA and 2.0 mM SUL-PLGA. Both solutions were prepared in Chloroform-d stabilized with silver (99.8% isotopic purity, Cambridge Isotope Laboratories, Inc., Cambridge, MA, USA). The chemical shifts of δ = 7.26 and δ = 77.23 (3) were used as a reference.

### 3.5. SUL Functionalization Degree on the Carboxylic Groups of PLGA

The functionalization degree (FD) of the PLGA carboxylic group to amide groups was assessed by comparing the elemental analysis of the PLGA and SUL-PLGA and using the ^13^C-NMR integration of the SUL-PLGA peaks. The elemental analysis was performed on a Perkin-Elmer 2400 CHN analyzer. The results of the elemental analysis were used to determine the FD (the percentage of carboxyl groups replaced in each PLGA monomer by SUL ligand). The FD was determined as described in the literature [69].

### 3.6. Preparation of 5-FU-Loaded PLGA and SUL-PLGA Nanoparticles

The 5-FU-loaded PLGA NPs (5-FU-PLGA) and SUL-PLGA NPs (5-FU-SUL-PLGA)) were prepared using the nanoprecipitation method [70,71]. In this method, the organic phase is comprised of a polymer dissolved in a polar organic solvent, such as acetone or acetonitrile and the aqueous phase consists of an aqueous solution with the dissolved drug. The procedure is the dispersion of the organic phase in the aqueous phase under magnetic stirring, causing a spontaneous emulsification due to miscibility of both phases [72]. Then, the organic solvent is removed by reduced pressure, forming the nanoparticles, as a result of rapid diffusion of the organic phase through the aqueous phase [72,73,74]. Briefly, polymer (PLGA or SUL-PLGA, 100 mg) was dissolved in acetone (8 mL) and stirred for 2 h at room temperature. 5-FU (30 mg) was dissolved in aqueous solution (25 mL) using different concentrations of Pluronic (0.1% to 1% w/v). However, the best results were obtained using 0.1% w/v of Pluronic F-68. PLGA or SUL-PLGA solutions were added to the water solution. The NPs were formed immediately, and the solvent was removed through overnight evaporation at room temperature. The resulting suspension was centrifuged for 30 min at 18,000 rpm and washed three times with water before the dry-freezing process. The supernatant was recovered for 5-FU quantification.

### 3.7. Particle Size Analysis—Dynamic Light Scattering (DLS) Analysis

The particle sizes (e.g., the average hydrodynamic diameter) of 5-FU-SUL-PLGA, SUL-PLGA,5-FU-PLGA and PLGA were determined using a Malvern Zetasizer Nano ZS instrument (Malvern Instruments, Malvern, UK) and polyethylene square cells. The sample suspensions were exposed to monochromatic light (10 mW He-Ne laser, wavelength 632.4 nm) and scattered light intensity was measured at 90°. The hydrodynamic diameters (D_h_) were calculated from the average of three independent measurements that were the mean of 15 counts. The reported D_h_ is the average of three independent titrations, and the error bars represent the standard deviation (SD).

### 3.8. Scanning and Transmission Electron Microscope

The shape and surface morphology of the NPs were examined using scanning electron microscopy (SEM) (FEG with a nanofabrication system FIB—Quanta FEG 3D FEI, Hillsboro, OR, USA). An appropriate sample of NPs was mounted on metal (aluminium) stubs, using double-sided adhesive carbon tape. The samples were sputter-coated with 5 nm gold for 5 min at 14 mA under an argon atmosphere for secondary electron emissive SEM and observed for morphology at an acceleration voltage of 2.5 and 10 kV.

Additionally, the shape and surface morphology of SUL-PLGA NPs at different degradation states were investigated using transmission electron microscopy (TEM) (Tecnai G2-12—Spirit Biotwin FEI—120 kV, FEI Company, Eindhoven, Netherlands). TEM was carried out to determine the surface characteristics of the NPs in aqueous medium using a 3-mm Forman (0.5% plastic powder in amyl acetate)-coated copper grid (300 mesh) at 60 kV.

### 3.9. Zeta Potential (ZP) Measurements

The zeta potential (ZP) values were obtained through electrophoretical mobility (EM) measurements using the Smoluchowski equation [75]. The EM was, in turn, determined with the Laser Doppler Micro-electrophoresis technique at a scattering angle of 173° using a Malvern Zetasizer Nano ZS apparatus. The zeta potential was reported as the average of three readings over twelve cycles.

### 3.10. 5-FU Encapsulation Efficiency (EE) and Drug Loading

The amount of 5-FU encapsulated in the PLGA NPs was quantified using UV-vis spectrophotometry and the determination of the 5-FU concentration in the remaining supernatant collected after the preparation of the NPs. Briefly, 1 mL of each of the supernatants, along with 1 mL standard solutions of 5-FU was individually transferred into test tubes and quantitatively determined by comparison with a standard curve. The absorption bands of 5-FU overlap those of the SUL to some extent; therefore, the absorption at wavelength 205 nm was used for the quantitative analysis. The determination of the entrapped drug quantity was performed by separating the NPs from the aqueous suspension with centrifugation at 18,000 rpm for 30 min. The 5-FU standard curves were obtained based on the absorbance measured using a UV-vis spectrophotometer (Shimadzu UV 240) at wavelength 205 nm.

### 3.11. 5-FU Release Kinetics

The release experiment was carried out in phosphate buffer (pH 7.4). First, 20 mg 5-FU-SUL-PLGA NPs was suspended in 1 mL phosphate buffer at 37 °C with horizontal shaking (100 rpm). For comparison, a release evaluation of 5-FU-PLGA was also performed. At predetermined time intervals, the suspension of NPs was centrifuged, and the supernatant collected for further 5-FU analysis. The NPs were resuspended in the same volume of fresh medium and incubated again under the same conditions. The amount of 5-FU released in each time interval was determined using a UV-vis assay as previously described for the measurement of encapsulation efficiency. The amount of 5-FU released in each sample was determined using a calibration curve; the reported values are the averages of two replicates (*n* = 2). The results of the *in vitro* drug release studies were tabulated and shown graphically as the cumulative % drug released *vs.* time.

### 3.12. Acid-Base Titration and Zeta Potential (ZP) Measurements

5-FU-loaded NPs (50 mg) were suspended in NaCl aqueous solution (150 mM; 10 mL) with 1 M NaOH (aq.) (200 µL). The basic 5-FU-loaded NPs suspensions were titrated by using 0.1 M HCl at room temperature (RT). The changes of pH and zeta potential were monitored in accordance with the method described in the literature [67].

### 3.13. In Vitro Cell Culture Studies

Two cancer cells lines, Caco-2 and A431, and two types of normal cells, a human fibroblast cell line and osteoblasts (primary culture), were used. The Caco-2 and A431 cell lines were cultured using a method described elsewhere [76,77]. Briefly, the Caco-2 and A431 cells were cultured in Dulbecco’s modified Eagle’s medium DMEM with 10% FBS, L-glutamine (2 mM), penicillin and streptomycin (100 IU/mL), and HEPES (25 mM), and maintained at 37 °C in a humidified incubator containing 5% CO_2_. The medium was changed 2 to 3 times a week. The cells were harvested with trypsin-EDTA after reaching 80% confluence. The fibroblast cells were grown in DMEM supplemented with 10% FBS and penicillin and streptomycin (100 IU/mL) in an incubator containing 5% CO_2_ at 37 °C. After reaching confluence, the cells were used for experiments on the 7th passage and seeded at a density of 10^5^ cells/mL in 96 wells plates [78]. The osteoblasts were isolated from the calvaria of 1–4 day old male Wistar rats obtained from the bioterium of the Institute of Biological Science, UFMG. These cells were used for this experiment according to the experimental protocol approved by the animal experiment committee of UFMG, Brazil (304/12) as demonstrated by [79].

### 3.14. Cytotoxicity Assays

The cytotoxicity of free 5-FU and 5-FU-loaded NPs was determined using an MTT assay. The cells were seeded in 96-well plates at a density of 10^5^ cells/well and incubated for 72 h before the assay. Then, the cells were incubated with free drug or drug loaded NPs for 72 h. The 5-FU concentration in the various formulations was 50.0, 25.0 and 12.5 µg/mL. Each 5-FU concentration was tested in six replicates in each of three separate experiments. The effect on cell viability of each formulation at different concentrations was expressed as a percentage by comparing the treated cells with cells incubated only with the culture medium. NPs without 5-FU were used to test the cytotoxicity of the blank. After 72 h of incubation with various treatments, cell viability was evaluated using an MTT assay based on the reduction of tetrazolium salt to formazan crystals in living cells. Approximately 10 μL MTT (5 mg/mL) was added to each well. Four hours later, cell morphology was analyzed using inverted optical microscopy, and formazan crystals were dissolved in 10% SDS in 0.01 M HCl. After incubation for 14 h, the optical density was measured at 570 nm. All quantitative results were obtained from hexaplicate samples. The data were expressed as the mean ± SD. The statistical analysis was carried out using ANOVA and Bonferroni’s post-test. A value of *p* < 0.05 was considered statistically significant.

## 4. Conclusions

We have developed novel 5-FU-SUL-PLGA NPs that provide the efficient delivery of 5-FU to Caco-2 and A431 cancer cells. Moreover, the SUL functionalization did not cause morphological or size differences in the particles, nor did it change the 5-FU solubility in the polymer core. The presence of SUL enhanced the cytotoxicity of the 5-FU-SUL-PLGA NPs when compared with 5-FU-PLGA NPs and had minimal effect on normal cells. Although further *in vivo* experiments must be conducted to confirm the higher cytotoxicity and selectivity of 5-FU-SUL-PLGA NPs to cancer cells, the development of 5-FU-SUL-PLGA NPs is a promising strategy for 5-FU formulation in the future.

## Supplementary Materials

Supplementary materials can be accessed at: http://www.mdpi.com/1420-3049/20/01/0879/s1.

## List of Compounds

5-fluorouracil: (PubChem CID: 3385)
Sulfadiazine: (PubChem CID: 5215)
poly(lactide-co-glycolide)—PLGA: (PubChem CID: 23111554)
1-Ethyl-3-(3-dimethylaminopropyl) carbodiimide: (PubChem CID: 15908)
N-hydroxysuccinimide: (PubChem CID: 80170)
