# Supplementary material for: Development of Sulfadiazine-Decorated PLGA Nanoparticles Loaded with 5-Fluorouracil and Cell Viability
Source: Molecules. 2015 Jan 8;20(1):879–99. doi: 10.3390/molecules20010879 (PMC6272719; doi:10.3390/molecules20010879)
Supplement: Supplementary file 1 [file molecules-20-00879-s001.pdf]

Supplementary Materials

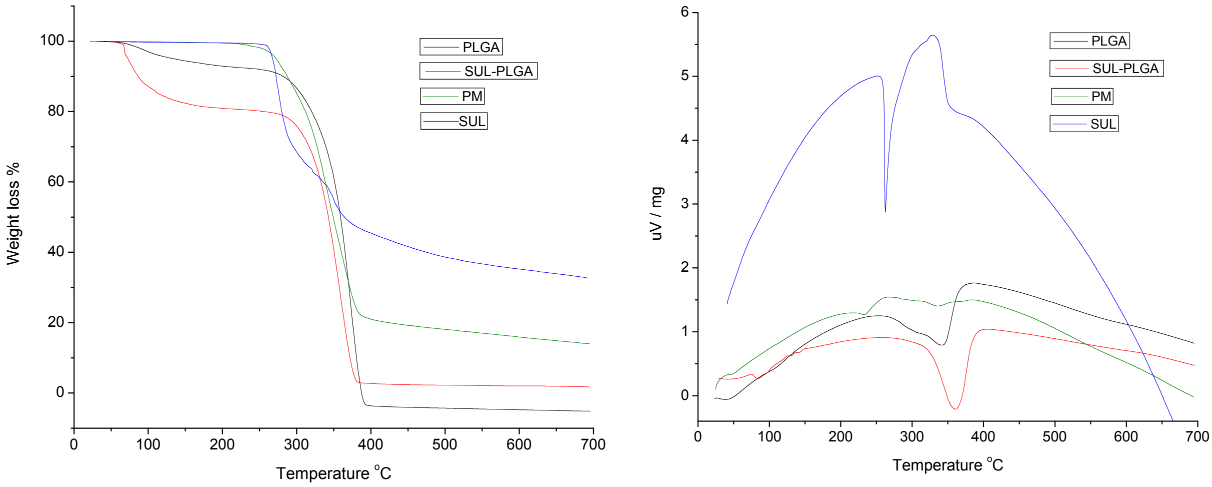

(a) **(b)**

Figure S1. TGA (**a**) and DTA (**b**) curves of PLGA (**black line**), SUL-PLGA (**red line**), Physical mixture of SUL-PLGA (PM—**green line**) and Sulfadiazine (SUL—**blue line**).

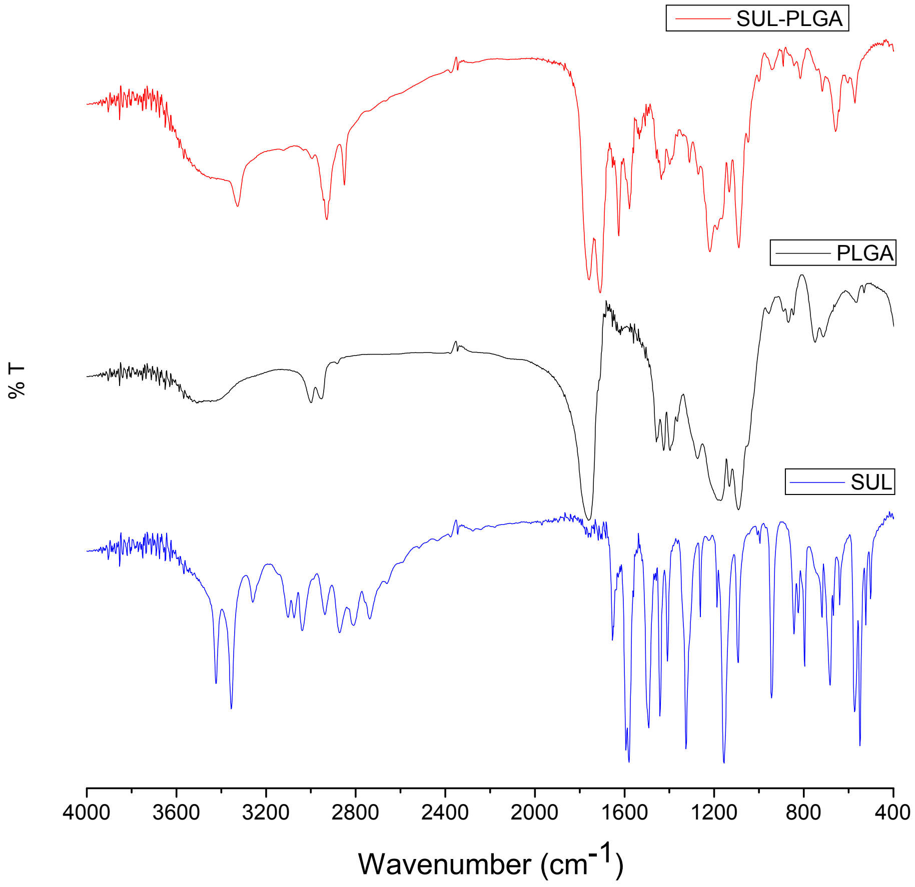

Figure S2. FTIR spectra of PLGA (**black line**), SUL-PLGA (**red line**) and SUL (**blue line**).

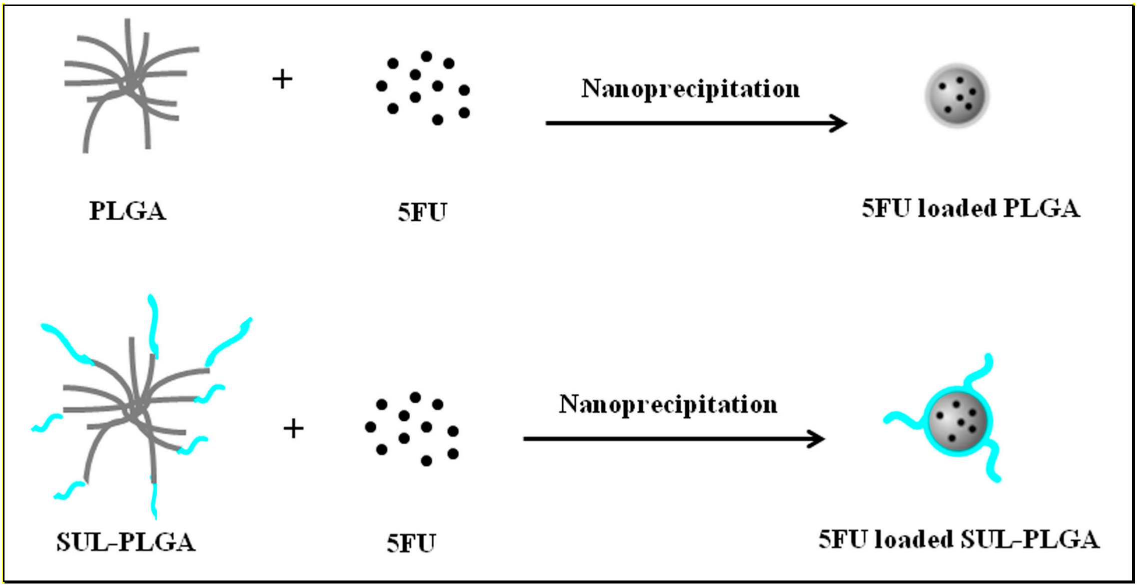

Figure S3. Schematic representation of the preparation of 5FU-loaded PLGA and SUL-PLGA NPs.

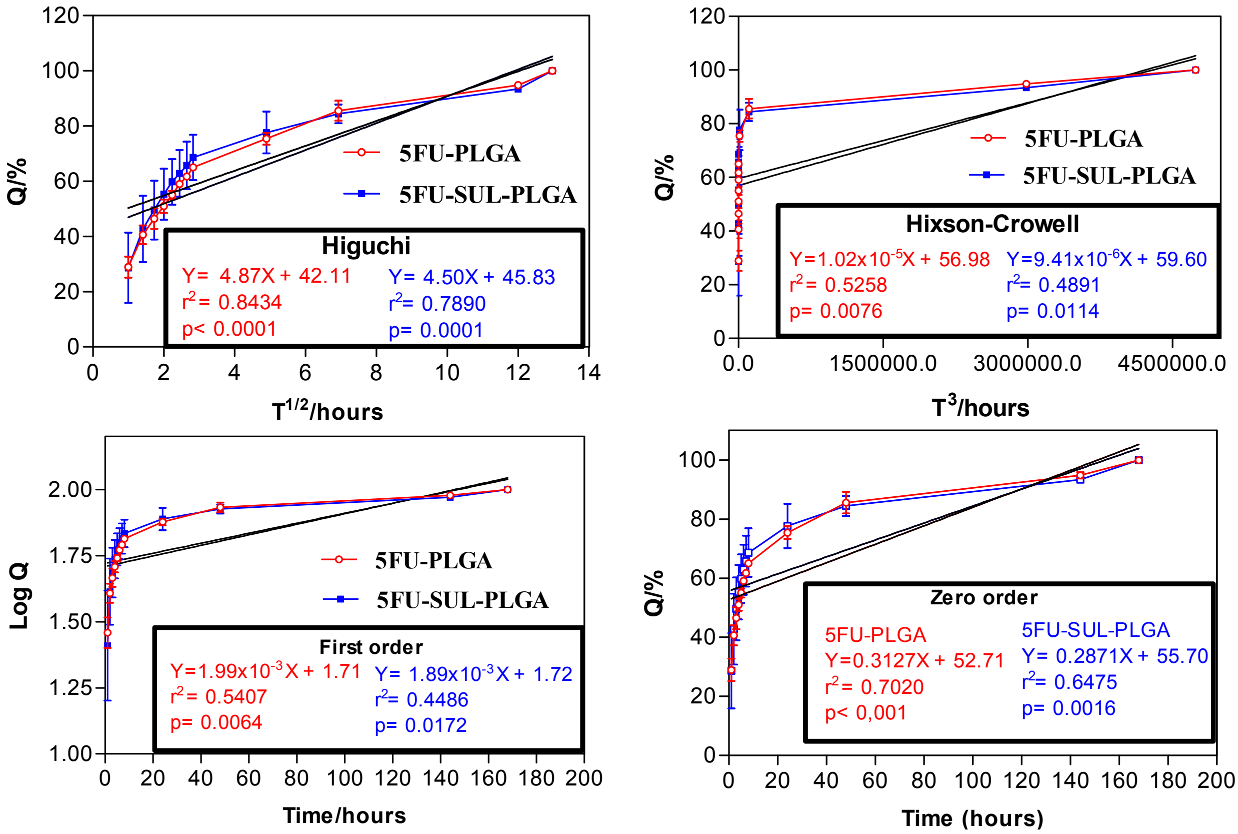

Figure S4. Release profiles of 5-FU from 5FU-PLGA and 5FU-SUL-PLGA *in vitro* in different models (vertical bar means average ± standard deviation, $n = 2$).

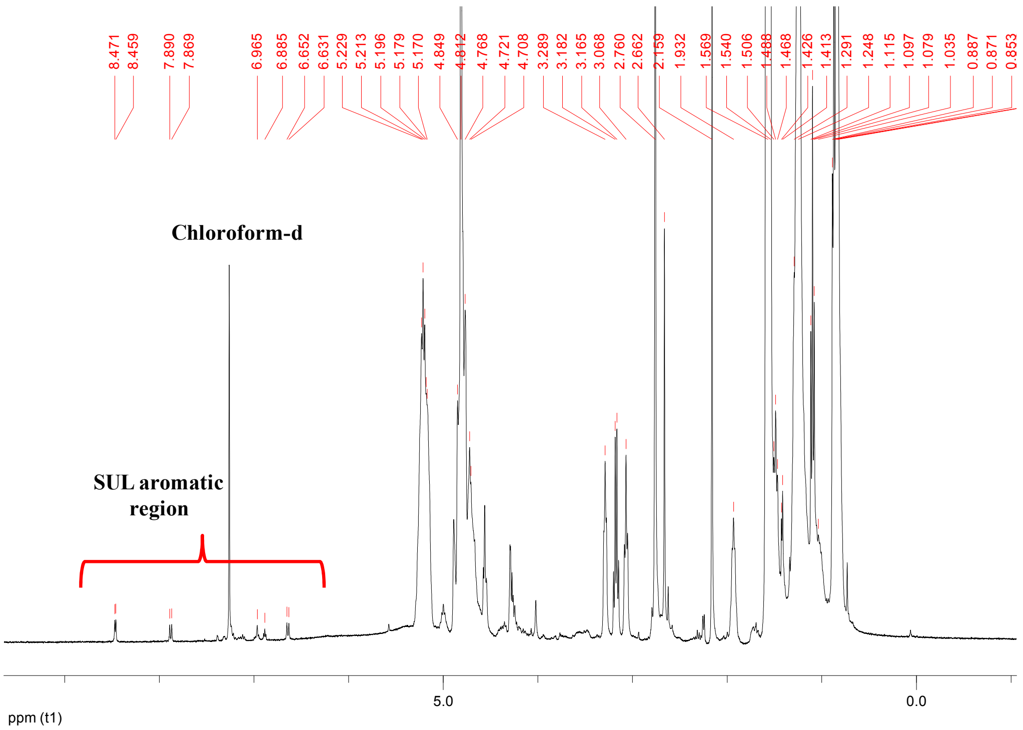

Figure S5. ^{1}H-NMR Spectrum SUL-PLGA (Chloroform-d, 400 MHz).

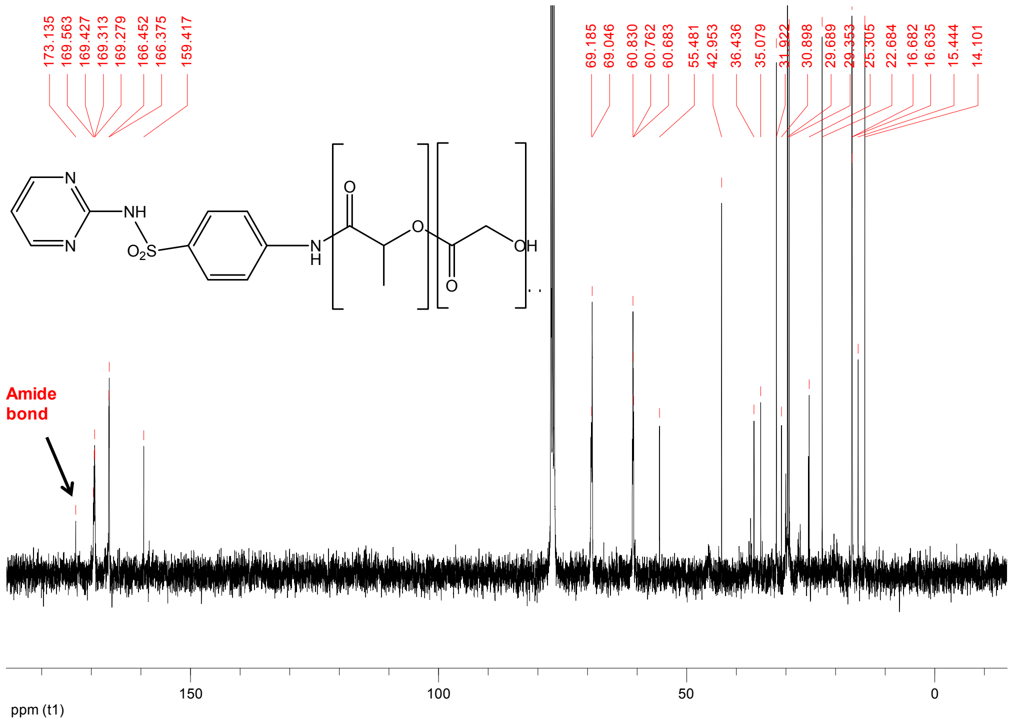

Figure S6. ^{13}C-NMR Spectrum SUL-PLGA (Chloroform-d, 400 MHz).

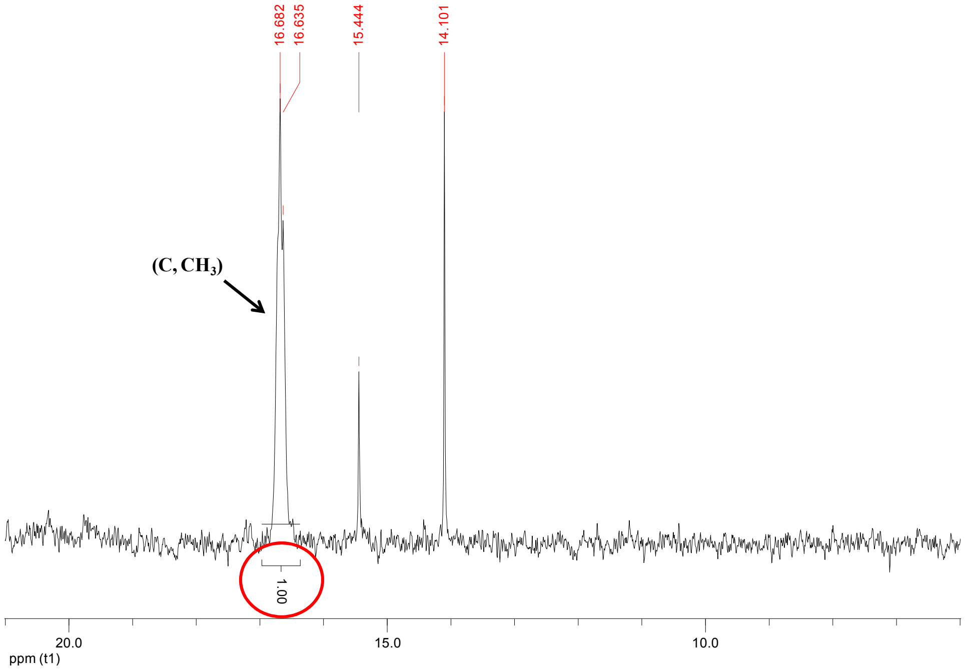

Figure S7. Expansion of the region of C, CH_3 of PLGA, with the integration of the peak in the ^{13}C-NMR SUL-PLGA spectrum (Chloroform-d, 400 MHz).

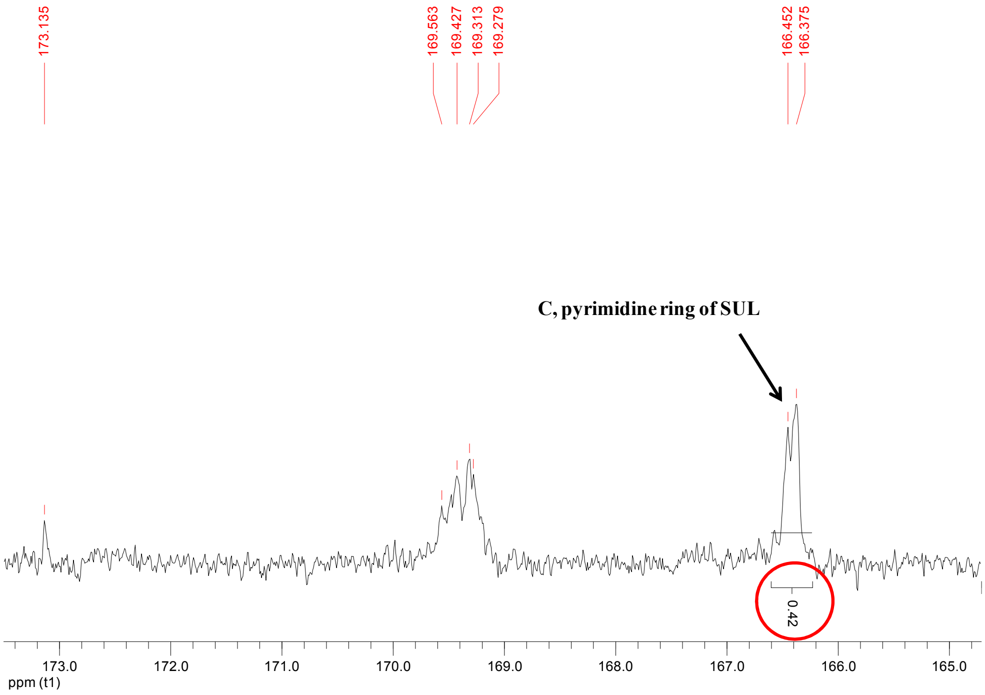

Figure S8. Expansion of the region of C, pyrimidine ring of SUL, with the integration of the peak in the ^{13}C-NMR SUL-PLGA spectrum (Chloroform-d, 400 MHz).

$$EE = (\text{weight of drug added} - \text{weight of free drug in supernatant/weight of drug added}) \times 100 \quad (1)$$

$$LC = (\text{weight of drug in NPs/weight of nanoparticles}) \times 100 \quad (2)$$
